# Teachers' Perception of Student Coping With Emergency Remote Instruction During the COVID-19 Pandemic: The Relative Impact of Educator Demographics and Professional Adaptation and Adjustment

**DOI:** 10.3389/fpsyg.2021.648443

**Published:** 2021-04-22

**Authors:** Magdalena Jelińska, Michał B. Paradowski

**Affiliations:** ^1^Institute of Applied Linguistics, University of Warsaw, Warsaw, Poland; ^2^Institute of Linguistics, University of Silesia, Sosnowiec, Poland

**Keywords:** educational psychology, COVID-19 pandemic, evaluation, emergency remote teaching, school closure, distance learning, perception of student coping, professional adaptation

## Abstract

The outbreak of the COVID-19 pandemic has upended lives and thrown the taken for granted into disarray. One of the most affected groups were teachers and students, faced with the necessity of school closures and—where logistically feasible—an urgent shift to emergency remote instruction, often with little prior notice. In this contribution, based on an online survey involving participants from 91 countries, we offer a perspective bridging the two groups, by investigating the role of teachers' demographics and professional adaptation to emergency remote teaching in their perception of how their students were coping with the novel situation. The resultant model explains 51% of variance, and highlights the relative weights of the predictor variables. Given the importance of teacher perceptions in the effectiveness of their instruction, the findings may offer valuable guidelines for future training and intervention programs.

## Introduction: Background Literature

In the wake of the COVID-19 pandemic, early 2020 saw a global suspension of face-to-face classes and large-scale school closures in an attempt to curb the viral transmission, impacting over 90% of the world's student population (UNESCO, 2020). In order to ensure continuity of education, where logistically possible institutions transitioned to emergency remote instruction, usually with little time given for preparation. The transition and implementation of the new teaching and learning format have posed numerous risks, problems, and challenges to both teachers and students (Bao, 2020; Cachón-Zagalaz et al., 2020; Hiraoka and Tomoda, 2020).

Current scholarship on emergency remote instruction during the COVID-19-induced school closures tends to focus either on teacher or student populations. The University of Houston's (2020) report summarizing the faculty's perceptions regarding the transition revealed significant variation in terms of the implementation of technology tools and of the mode of instruction. A survey by Quality Matters and Eduventures^®^ Research (Legon et al., 2020) carried out among chief online officers at colleges and universities reported that while most believed the pivot to remote teaching to be a logistic success, most simultaneously admitted at least a measure of difficulty, citing low levels of faculty and student preparedness. A report published by Ohio State University (Jaggars et al., 2020, p. 25) discovered among others that faculty who taught their courses in real-time rated online teaching challenges less negatively than those who used an asynchronous delivery format. A survey deployed by eLearning Researc Practice Lab Indiana University Pervasive Technology Institute (2020) found that two-thirds of the instructors felt disconnected from their students and that it was more difficult to teach, while three quarters of the students felt they had lost touch with their IU community; a similar number declared it took them more effort to complete their course assignments after the transition. A study carried out among university students at a Greek university (Karalis and Raikou, 2020) found that the majority of the students surveyed experienced negative emotional states such as stress, anxiety, and sadness at the announcement of university closure, but that these decreased once online classes had started. Thematic analyses of the responses revealed the important role of the teachers in this positive shift. Watermeyer et al. (2020) found that the majority of the academic teachers in their sample felt confident or strongly confident in their ability to carry out online teaching and assessment, and considered their institutions to be supportive in facilitating the move to online delivery. Li et al. (2020) assessed the impact of the outbreak on stress, poor mental health, and poor sleep quality symptoms among college students in Guangdong. Wilczewski et al. (2021) investigated the psychological and academic effects of learning online among international students enrolled at the University of Warsaw. Alemany-Arrebola et al. (2020) analyzed the impact of trait- and state-anxiety on university students' perception of academic self-efficacy in Spain. Zaccoletti et al. (2020) investigated the decrease in Italian and Portuguese primary and lower secondary school pupils' motivation from the perspective of the parents.

A sudden forced transition to remote teaching constitutes an important factor that may influence students' handling of this unprecedented educational challenge, as well as teachers' perception of students' coping with remote learning. Perceptions of students' abilities guide teachers' choices of instructional methods (Biddle and Anderson, 1986; Snow, 1994; Martin, 2006; Hardré and Sullivan, 2009; Cho and Shim, 2013). Those who perceive their students' achievement, motivation, work habits, and class activity negatively have lower learning expectations (Rubie-Davies, 2010). Highly consequential for the efficiency of the teaching process and student achievement is also teachers' perception of their own competencies (Tucker et al., 2005; Guo et al., 2012; Miller et al., 2017). Numerous studies indicated that teacher self-efficacy positively influences beliefs about teaching (Tschannen-Moran and Woolfolk-Hoy, 2001; Skaalvik and Skaalvik, 2007; Cho and Shim, 2013), modes of instruction, and students' outcomes (Zee and Koomen, 2016). Teachers' perception of their own self-efficacy is also related to how they meet obstacles and challenges (Pajares, 1996; Tschannen-Moran et al., 1998; Woolfolk Hoy et al., 2005). Instructors who feel more efficient and competent themselves set higher expectations for their students (Midgley et al., 1995; Wolters and Daugherty, 2007; Cho and Shim, 2013), but simultaneously are more positive and responsive to them (Gibson and Dembo, 1984) and tend to provide them with more support promoting a good learning atmosphere (Bru et al., 2002; Rubie-Davies, 2007, 2010; Sakiz et al., 2012; Holzberger et al., 2013, 2014; Guo et al., 2014).

The results of the above studies refer to the educational process under normal conditions of on-site and face-to-face classes. Little is known about whether they will be replicated in the case of emergency remote teaching. Existing studies concerning online teaching or learning focus mainly on identifying the most significant technical skills such as those needed to use the specific software application, or on dealing with difficulties with particular software and using it in student assessment of learning, as illustrated for instance in Hampel and Stickler's (2005) skills pyramid (see also Livingston and Condie, 2006; Compton, 2009). However, research has not yet attempted to investigate how teachers' circumstances, behaviors, and attitudes affect their perception of students' coping with online delivery. Moreover, remote learning entails specific problems that may influence teaching and learning quality and effectiveness, such as the deficit of “live,” “face-to-face” contact felt by both students and teachers (e.g., Stodel et al., 2006; Arroyo et al., 2015; Barnard-Ashton et al., 2017; Janse van Rensburg, 2018;^1^); lack of adequate technological affordances to efficiently deliver the program, provide support to participants, and satisfy their learning needs; and inadequate teacher and student competencies to use the technological solutions (Garrison et al., 1999; Pawan et al., 2003; Livingston and Condie, 2006). These deficits can result in increased feelings of insecurity, confusion, and threat among learners, as well as feelings of doubt and fear of failure among teachers (Arroyo et al., 2015; Janse van Rensburg, 2018) influencing how they perceive their students and, in consequence, how they teach them.

Thus, teachers' sense of competence, which is related to the notion of self-efficacy (Bandura, 1997), may facilitate adaptation, enhancing their ability to deal with challenges and difficulties related to this novel teaching context. It may also be linked to the degree to which the teachers support their students in the new learning context as well as in the COVID-19 pandemic social context, if it shapes expectations toward the students, adjustment of instruction to this new situation and the students' abilities, evaluation of their activity, as well as potential difficulties with assessment. All these factors seem to be related to if not determine how teachers perceive students' coping during the transition to remote instruction. The social projection hypothesis (Krueger and Acevedo, 2005; Krueger et al., 2006; Krueger, 2007) permits the conjecture that teachers' own sense of competence and how they cope in this particular educational context as well as their satisfaction with remote teaching and perception of its effectiveness may be automatically projected onto and attributed to students.

The aim of this study is thus to investigate the ways in which teachers' perception of how their students were coping with the novel situation was affected by their professional adaptation to emergency remote teaching (ERT; Hodges et al., 2020), following the social projection hypothesis (Krueger and Acevedo, 2005; Krueger et al., 2005; Krueger, 2007), as well as sociodemographic variables. The study is guided by the following exploratory research questions:

RQ1: To what extent—if any—does teachers' perception of students' coping vary depending on sociodemographic factors such as (1a) teachers' gender, (1b) teachers' age, (1c) length of teaching experience, and (1d) level of education handled?
RQ2: To what extent—if any—does teachers' perception of students' coping vary depending on their attitudes toward remote teaching based on (2a) perception of remote teaching effectiveness, (2b) prior experience with remote instruction, (2c) mode of remote instruction, i.e., synchronous vs. asynchronous classes, and (2d) appraisal of the relative situational impact on teachers and students (i.e., whether one of these group is more affected)?
RQ3: Which of the indicators of teachers' professional adaptation to emergency remote instruction are associated with teachers' perception of student coping with online classes, and to what extent?
RQ4: Which of the indicators of teachers' professional adaptation to emergency remote instruction predict their perception of student coping with online classes?
RQ5: What is the relative contribution of the respective predictors and to what extent does each of them determine teachers' perception of student coping with online classes? Which of them have the greatest influence on how teachers perceive their students' learning?

To this end, from April until September 2020, we carried out a custom-made multinational survey.^2^

## Materials and Methods

### Participants

From April through September 2020, a total of 6,582 educators participated in a study probing teachers' adaptation to emergency remote instruction during the COVID-19 epidemic. Of these, 1,944 completed the questionnaire and met the inclusion criteria. Approximately 40% represented higher education institutions, teaching at a university, graduate school, or community college; 24.3% taught at secondary and 29.2% at K-primary education levels. The teachers came from 6 continents and 91 countries and autonomous territories, with almost equal proportions representing Europe (41.6%) and North America (40.6%). Their reported mean age was 43.6 years (SD = 11.9). More than half (51%) were aged between 25 and 45; 44% declared to be over 45 years old; 83% were female.

Most of the participants had been teaching their subject for more than 5 years. Almost 62% declared to teach classes in real time (synchronously) during the COVID-19 pandemic, although as many as 79.1% had no prior experience with this mode of instruction. More than 62% of all the respondents found remote teaching (whether synchronous or asynchronous) less effective than face-to-face classes; 53.2% estimated that the pandemic situation had affected teachers and students equally, whereas 33.1% found students to be in a worse situation than the teachers. The participant sociodemographics are presented in Table 1.

**Table 1:** Sociodemographic characteristics of the participants (*N* = 1,944).

	**Frequency (*n*)**	**Percent (%)**
**Stage of education handled**		
K-primary	568	29.2
Secondary	472	24.3
Tertiary	772	39.7
Other	27	4.2
Not reported	51	2.6
**Continent**		
**Europe**	809	41.6
(Austria, Belgium, Belarus, Bulgaria, Czech Republic, Croatia, Cyprus, Denmark, Finland, France, Georgia, Germany, Greece, Hungary, Ireland, Italy, Jersey, Lithuania, Luxembourg, Malta, Montenegro, Netherlands, Norway, Poland, Portugal, Romania, Russia, Serbia, Slovakia, Spain, Sweden, Switzerland, United Kingdom, and Ukraine)		
**North America**	789	40.6
(Bahamas, Canada, Costa Rica, Mexico, Puerto Rico, and USA)		
**Asia**	200	10.3
(Bahrain, Bangladesh, Brunei, Cambodia, China, Hong Kong, Macao, India, Indonesia, Iraq, Israel, Japan, Jordan, Kuwait, Laos, Lebanon, Malaysia, Nepal, Oman, Pakistan, Palestine, Philippines, Qatar, Saudi Arabia, South Korea, Sri Lanka, Taiwan, Thailand, Turkey, United Arab Emirates, and Vietnam)		
**Oceania**	72	3.7
(Australia, Fiji, Kiribati, and New Zealand)		
**South America**	38	2.0
(Argentina, Brazil, Chile, Colombia, Peru, Trinidad and Tobago, and Uruguay)		
**Africa**	36	1.9
(Algeria, Egypt, Kenya, Lesotho, Malawi, Mauritius, Morocco, Senegal, South Africa, and Tunisia)		
**Age groups (years)**		
<25	95	4.9
25–35	397	20.4
36–45	595	30.6
46–55	533	27.4
56–65	282	14.5
>65	40	2.1
Not reported	2	0.1
**Gender**		
Female	1,610	82.8
Male	320	16.5
Not listed/non-binary	14	0.7
**Experience teaching the subject**		
<5 years	615	31.6
6–15 years	620	31.9
16–25 years	473	24.3
26–35 years	178	9.2
>35 years	57	2.9
Not reported	1	0.1
**Remote teaching mode**		
Synchronous	1,202	61.8
Asynchronous	742	38.2
**Prior experience with remote teaching**		
Lack of experience	1,537	79.1
Prior experience	407	20.9
**Perceived effectiveness of remote teaching vis-à-vis F2F instruction**		
Less efficient	1,217	62.6
Equally efficient	526	27.1
More efficient	201	10.3
**Appraisal of relative situational impact**		
Students affected more than teachers	644	33.1
Teachers and students affected equally	28	53.2
Teachers affected more than students	113	5.8
Not reported	153	7.9

### Measures

To verify how teachers adapt to and cope with emergency remote teaching, we designed a custom-made online survey (see Supplementary Material). It comprised 441 items concerning respondents' sociodemographics, the circumstances surrounding their transition to emergency remote instruction, personal experiences, behaviors, attitudes, feelings, physical and mental health, as well as their personality traits. Psychological constructs were measured with 23 short scales developed from IPIP items and inspired among others by in-depth analyses of Brief COPE (Carver, 1997), Life Orientation Test—Revised (Scheier et al., 1994), Five-Dimensional Curiosity Scale Revised (5DCR; Kashdan et al., 2020), Individual Adaptability I-ADAPT-M (Ployhart and Bliese, 2006), and Grit Scale (Duckworth et al., 2007). Due to the rather general nature of these existing questionnaires, which does not permit capturing more situation-oriented circumstances, we developed custom-made scales, single-item indicators, as well as open-ended questions. The content of these items was consulted with academics with experience in the field and discussed/piloted with teachers and students.

In this article, to analyze how teachers' professional adaptation to emergency remote instruction contributes to their perception of student coping, we focus on five short scales and nine specific single-item indicators complemented by sociodemographic factors such as gender and education stages handled. Two of the designed scales assessed aspects of teacher adaptation to ERT such as adjustment of instruction and evaluation uncertainty/issues, one scale measured perceived student coping, whereas the other two scales investigated self-perception of supportive teaching and sense of competence.

*Instructional adjustment* was measured with three items assessing the extent to which the respondents had modified their teaching mode and material (e.g., “During this epidemic, I have felt that I have to alter not just the medium and method, but also the content of my classes”) as well as changed their evaluation (e.g., “I have eased the grading scheme”). The items in this and all the remaining scales were answered on a six-point Likert scale ranging from 1 (completely disagree) to 6 (completely agree). The internal consistency of this scale was satisfactory (Cronbach's α = 0.70, McDonald's ω_h_ = 0.68, Guttman's λ_6_ = 0.59, Reykov's ρ = 0.89).

*Evaluation uncertainty* was measured with a three-item scale assessing the extent to which teachers experienced difficulties in evaluating students' activity (e.g. “I find it difficult to evaluate students' activity during online classes”) and verifying progress (“I am unable to verify whether the students are learning”) as well as doubts about the integrity of the assignments and examinations (“I am anxious that some students may be using the situation to cheat in their assignments/exams”). In the current study, its internal consistency reached Cronbach's α of 0.73, McDonald's ω_h_ = 0.75, Guttman's λ_6_ = 0.68, and Reykov's ρ = 0.98.

*Perceived student coping* was assessed with four items measuring teachers' perception of the extent to which students coped with the transition to remote learning (e.g., “I feel that some of my students have been left behind/fallen through the cracks in the shift to remote teaching”), reacted positively to remote teaching (“My students have responded positively to my remote teaching”), and experienced difficulties such as lack of concentration and/or attention (“The students have trouble with concentration and staying focused during the online classes”). Three of the items were reverse worded, indicating issue occurrence. The scale reported a satisfactory internal consistency of Cronbach's α = 0.72, McDonald's ω_h_ = 0.74, Guttman's λ_6_ = 0.69, and Reykov's ρ = 0.94.

*Supportive teaching* was measured with a five-item scale gauging to what extent teachers perceived themselves as motivating (“I can talk my students into learning”), supporting students' activity (“I am good at helping people work well together”), inclusive (“I try to make sure everyone in a group feels included”), building their relation with the students/class on trust and security (“I trust my students”), as well as fostering student well-being (“I try to make my group members happy”). The scale showed good internal consistency of Cronbach's α, McDonald's ω_h_ and Reykov's ρ = 0.73, and Guttman's λ_6_ = 0.71.

*Sense of competence* was measured with an eight-item scale assessing the extent to which teachers felt they had knowledge and skills necessary to face challenging or demanding tasks (e.g., “I know how to get things done” and “I like to work on tasks that require a great deal of skill”), preparedness (e.g., “I am always prepared”), having multitasking ability (“I can manage many things at the same time”), being tech-savvy (“I am a confident user of new technologies”), and lifelong training in their professional skills (“I continually train to keep my teaching skills and knowledge up-to-date”). In the current study, the scale reported good internal consistency of Cronbach's α and McDonald's ω_h_ = 0.76, Guttman's λ_6_ = 0.74, and Reykov's ρ = 0.67 and correlated positively with supportive teaching (*r* = 0.47, *p* < 0.05).

The scales were distinguished on the basis of exploratory factor analysis (EFA) preceded by an analysis of inter-item correlations. As the criterion for item selection, we followed the recommendation by Clark and Watson (1995) that items should ideally correlate between 0.15 and 0.50. Using maximum likelihood estimation with standardized varimax rotation, we identified five factors with eigenvalues exceeding 1.0 (from 4.4 for the first factor to 1.04 for the fifth factor). All loadings but two were greater than 0.40. All the factors accounted for 52% of total variance explained. The EFA factor loadings of all the items are presented in Appendix Table 6a. The model of these scales was subsequently validated through confirmatory factor analysis (CFA) using the SEPATH module in the STATISTICA software. For this purpose, we applied the maximum likelihood method, which resulted in the same five-factor structure. The comparative fit index (CFI; Bentler, 1990) estimates how much the non-centrality parameter is reduced when moving from an unstructured baseline model (i.e., one with the worst fit) to the considered model. Values of at least 0.90 indicate a good fit of the model. The root mean square error of approximation (RMSEA) based on the non-centrality parameter estimates the difference between the hypothesized model and the perfect model. A well-fitted model is indicated by values lower than 0.05 (Browne and Cudeck, 1993). The standardized root-mean-square residual (SRMR) reflects the discrepancy between the reproduced and the observed correlations. As the cutoff criterion, Hu and Bentler (1998) recommended a value of 0.08, with higher values indicating poorer fit to the data and values lower than 0.05 indicating an excellent fit. Both RMSEA and SRMR are the absolute fit indices. In this study, the CFA fit indices indicated satisfactory fit for the model: χ^2^(220) = 352.74, *p* = 0.001—given that χ^2^ is highly sensitive to large sample sizes and leads to the rejection of models with the simplest misspecification, we also examined the goodness-of-fit index GFI = 0.900, CFI = 0.904, RMSEA = 0.046, and SRMR = 0.061. They provided confirmatory evidence for the factor structure.

The single-item indicators were used to assess other aspects related to educators' perception of the students during the remote teaching period, such as *student activity evaluation* (“Thanks to the change, some students who used to be passive have now become more active”) and *overly demanding expectations* (“Now my students ought to be able to do more because they have more time”). They also allowed measuring COVID-19 situation-related aspects of teaching such as *reassuring attitude* (“I try to reassure my students during these times”), *perceived initial ability to teach remotely* (“I felt confident in my ability to teach remotely when I was told to do so”), *prior experience with remote teaching* (“I had been teaching e-learning, blended courses, MOOCs or webinars before this epidemic”), and *mode of remote instruction* (synchronous vs. asynchronous). Finally, some single-item indicators were used to measure *perceived remote teaching effectiveness, satisfaction with online teaching software/solutions* (“Do you like the tools/software/platform you have been using to teach the online classes?”), and the *appraisal of the relative situational impact* of the COVID-19 pandemic on teachers and students in the educational context. This item measures instructors' perception of how the situation influences different stakeholders of the education process: as affecting teachers more than students, students to a greater extent than teachers, or influencing both groups to a similar extent. In addition to single-item indicators, we took into account basic sociodemographic information such as teachers' age, gender, and education stage handled.

One-item measures are often criticized for their low reliability and estimation difficulty, as well as vulnerability to the response style effect compared with longer scales, but these arguments are not always sufficiently justified (Wanous and Reichers, 1996; Konstabel et al., 2012; Jovanović and Lazić, 2020). In psychometric theory, the longer the scale, the lower the measurement error and thus the higher scale reliability. However, with an increasing number of items, respondents may pay decreasing attention to their responses, which reduces the quality of the information obtained (Gogol et al., 2014). Moreover, Wanous and Reichers (1996) proposed a procedure based on a formula for the correction for attenuation which allows measuring the reliability of single-item indicators. In this study, we applied the comprehensive single item approach (CSI) proposed by Konstabel et al. (2012, 2017). The CSI assumes that the content validity of a one-item indicator is preserved when this item has a comprehensive content and is newly written instead of being selected from a longer scale. This approach is based on the assumption that every individual has self-knowledge and is able to characterize it if a given construct is substantially simple, unambiguous, or narrow to be comprehensible to the respondent (Wanous and Reichers, 1996; also Loo, 2002). This means that the CSI should not be applicable to more complex traits or dispositions. Thus, in this research, it was only employed with general and more homogeneous variables. Numerous single-item measures have successfully been used in studies measuring self-esteem (Robins et al., 2001), self-efficacy (Hoeppner et al., 2011), job satisfaction (Wanous et al., 1997; Nagy, 2002), burnout (West et al., 2012), psychosocial stress (Littman et al., 2006), life satisfaction (Jovanović and Lazić, 2020), the need to belong (Nichols and Webster, 2013), ability ratings (Rammstedt and Rammsayer, 2002), and even personality facets (Rammstedt and John, 2007; Denissen et al., 2008; Konstabel et al., 2017). In the field of education, they have been used to measure teaching effectiveness (Wanous and Hudy, 2001), academic anxiety and academic self-concept (Gogol et al., 2014), among others. Single-item indicators are appreciated as they allow reducing survey time, are highly flexible, easily adjust to new contexts, and yield more generalizable research results that are easier to interpret. They also protect against item redundancy, which may evoke respondent frustration, mental fatigue, and boredom (Nagy, 2002; Konstabel et al., 2012, 2017; West et al., 2012; Gogol et al., 2014). Gardner et al. (1998) posit that in some contexts single-item general indicators of psychological constructs may be better than a set of responses to numerous construct facets. They are particularly useful in large-scale surveys that cover numerous variables (Nagy, 2002; Konstabel et al., 2012, 2017; West et al., 2012).

### Procedure

The custom-made questionnaire was active from April until September 2020 on a commercial survey software platform (in order to facilitate responses from countries where solutions such as Google Forms are inaccessible without a VPN). The participants were recruited based on a snowball sampling technique using several channels including the researchers' direct personal contacts, mailing lists and websites of professional associations, as well as thematic groups and pages on social media. The respondents were informed about the purpose of the survey and they participated voluntarily. The protocol had received IRB approval.

A prerequisite to take part in the study was having transitioned from regular face-to-face classes to online teaching as part of the response to the COVID-19 epidemic. The opening filter question excluded more than 13% of the initial survey takers, who either continued teaching face-to-face or had already been teaching online before the school closures happened.

### Data Analysis

In order to answer RQs 1a–d and 2a–d, the relationships between the variables, based on the significant differences, were analyzed on the basis of independent-sample *t* tests and one-way ANOVAs. To find the answer to RQ3, continuous variables were characterized by means of Pearson's correlation coefficient and categorical variables by Spearman's ρ. To verify the predictors of perceived student coping with remote teaching, we used STATISTICA's General Regression Models (GRM) module applying the methods of the general linear model, which allows building models combining categorical and continuous predictor variables (analysis of covariance design). In contrast with (multiple) regression models applicable only to continuous variables, the general linear model permits the analysis of any ANCOVA or MANCOVA design that includes both categorical (e.g., gender) and continuous predictor variables as well as a wide variety of different types of design. Analysis of covariance (ANCOVA) is defined as a general linear model, and combines at least one categorical predictor (one-way ANOVA) and continuous predictors (linear regression). In all general linear models, the dependent variable(s) is/are always continuous. As in the case of regression, the use of ANCOVA depends on five assumptions: normality of residuals, homogeneity of variance, homogeneity of regression slopes, linearity of regression, and independence of error terms (Garson, 2012; Philippas, 2014). In this study, a general linear model was the most suitable statistical tool to find the answers to RQ4, allowing to take into account both categorical and continuous predictors. The answer to RQ5 utilizes forward selection, based on adding the most statistically significant variables to the model until there are no more such variables meeting the entry criteria and a satisfactory regression equation has been found. The significance level was set at 0.001 for ANCOVAs and at 0.05 for the remaining analyses. Effect sizes are reported with Cohen's *d* for the *t* test and η_p_^2^ for ANOVA, respectively.

The linearity assumption was examined via a visual inspection of scatterplots showing that the variables and the residuals of the regression (i.e., the errors between the observed and the predicted values) were normally distributed. The variance inflation factor (VIF) not exceeding 1.6 and tolerance ranging from 0.64 to 0.94 indicated lack of multicollinearity. The lack of collinearity was also confirmed by a matrix of Pearson's bivariate correlations among all the predictors. A visual analysis of a scatterplot of residuals vs. predicted values indicated that the assumption of homoscedasticity was satisfied as well.

## Results

To answer the first two research questions, i.e., whether and to what extent teachers' perception of students' coping varied depending on sociodemographic factors such as (1a) gender, (1b) age, (1c) length of teaching experience, and (1d) education stage handled as well as whether and to what extent it varied depending on the teachers' attitudes toward remote teaching based on (2a) perception of remote teaching effectiveness, (2b) prior experience with remote instruction, (2c) mode of remote instruction, i.e., synchronous vs. asynchronous classes, and (2d) appraisal of the relative situational impact on teachers and students (i.e., whether one of these group was more affected), we calculated Student's *t* test and ANOVA, respectively. Table 2 presents significant differences for teachers' perception of student coping with remote learning. The results indicate that the teachers differed in their perception among several dimensions.

**Table 2:** Significant differences in teachers' perception of student coping with remote learning (*N* = 1,944).

	**Perceived student coping**						
	**M**	**SD**	**Effect size**	**95%CI**		***F* or *t***	**df**
**Gender**			***η***_**p**_^2^ **= 0.006**	**0.001**	**0.01**	***F*** = 5.71^*^	**2,1941**
Female	3.20^*^	1.01					
Male	3.42^*^	1.07					
Not listed/non-binary	3.34	1.03					
**Age group**			***η***_**p**_^2^ **= 0.11**	**0.08**	**0.13**	***F*** **= 74.28**	**3,1889**
<25 years	3.09	1.00					
25–35	3.20	1.03					
36–45	3.21	1.01					
46–55	3.26	1.03					
56–65	3.36	1.03					
>65 years	3.46	1.05					
**Education stage**			***η***_**p**_^2^ **= 0.004**	**0.0**	**0.008**	***F*** = 1.73^*^	**5,1907**
K-primary^bcd^	2.87^*^	0.99					
Secondary^acd^	3.04^*^	0.96					
Tertiary^ab^	3.58^*^	0.96					
Other^ab^	3.80^*^	1.02					
**Experience in teaching the subject**			***η***_**p**_^2^ **= 0.008**	**0.002**	**0.01**	***F*** **= 3.95**	**4,1938**
≤ 5 years	3.15	1.01					
6–15 years	3.29	1.02					
16–25 years	3.22	1.01					
26–35 years	3.32	1.11					
>35 years	3.60	00.97					
**Perceived relative effectiveness of remote teaching**			***η***_**p**_^2^ **= 0.20**	**0.018**	**0.23**	***F*** = 249.63^*^	**2,1941**
Less efficient	2.88^*^	0.89					
Equally efficient^e^	3.85^*^	0.90					
More efficient^e^	3.83^*^	1.07					
**Appraisal of relative situational impact**			***η***_**p**_^2^ **= 0.01**	**0.001**	**0.012**	***F*** **= 4.28**	**3,1940**
Students affected more than teachers	3.15	0.98					
Teachers and students affected equally	3.25	1.04					
Teachers affected more than students	3.41	0.99					
Not reported	3.42	0.98					
**Mode of remote instruction**			***d*** **= 0.64**	**0.55**	**0.74**	***t*** = 13.75^*^	**1,942**
Synchronous	3.48^*^	1.00					
Asynchronous	2.85^*^	0.95					
**Prior experience with remote teaching**			***d*** **= 0.27**	**0.18**	**0.36**	***t*** = 5.78^*^	**1,942**
Lack of experience	3.17^*^	1.02					
Prior experience	3.50^*^	1.02					

*Superscripts indicate significant pairwise differences based on Tukey's post-hoc test (p < 0.05) [for education stages: a—K-primary, b—secondary, c—tertiary d—other; for efficiency: e—less efficient]. The bold values always refer to the influence on perceived student coping of each respective superordinate category*.

Male teachers felt that students coped better (M = 3.42, SD = 1.07) compared with their female counterparts (M = 3.20, SD = 1.01). Differences also occurred across all three education stages: primary (M = 2.87, SD = 0.99), secondary (M = 3.04, SD = 0.96), and tertiary (M = 3.58, SD = 0.96).

Perception of student coping was also influenced by perception of remote teaching effectiveness. Those who found their remote instruction to be less efficient than face-to-face classes were convinced that students experienced more difficulties while learning online (M = 2.88, SD = 0.89), whereas their colleagues who estimated remote teaching as equally or more efficient did not deem it more difficult for their students (M = 3.85, SD = 0.90 and M = 3.83, SD = 1.07 respectively).

Student coping was also perceived differently among teachers who conducted synchronous and asynchronous classes. The former found students to cope better (M = 3.48, SD = 1.00), compared with teachers who did not teach in real time (M = 2.85, SD = 0.95).

Finally, differences in perception of student coping were also related to educators' prior experience with remote instruction. Teachers who had taught remotely before the pandemic estimated that their students were coping better and had fewer difficulties (M = 3.50, SD = 1.02), compared with teachers who had no prior experience with conducting online courses (M = 3.17, SD = 1.02).

Interestingly, differences in perception of student coping with remote teaching were unrelated to teachers' age, length of experience in teaching the subject, or appraisal of the relative situational impact on teachers and students.

Table 3 indicates the results of correlation analyses showing further relationships between indicators of teachers' professional and personal adaptation to ERT and perceived student coping with online learning, providing the answer to the third research question (RQ3). The way teachers perceive how their students cope with online courses is significantly and negatively correlated with teachers' evaluation uncertainty (*r* = −0.57) and instructional adjustment (*r* = −0.45). Moreover, the more effective the perception of remote teaching, the more the teachers felt students were coping with it (ρ = 0.44). Perception of student coping was also related to the education level handled, with more positive perceptions at higher education levels (ρ = 0.32). It also correlated with teachers' ease of evaluating their students' activity (*r* = 0.30). Finally, there is a trend suggesting that teachers who from the very outset had felt confident teaching remotely found that their students experienced fewer difficulties (*r* = 0.29).

**Table 3:** Pearson's *r*^[1]^ and Spearman's *ρ*^[2]^ correlation coefficients between indicators of teachers' professional adaptation to ERT and perceived student coping with online learning.

	**Perceived student coping**	***R*^**2**^**	**95%CI**	
Evaluation uncertainty^1^	−0.57^*^	0.33	0.29	0.36
Instructional adjustment^1^	−0.45^*^	0.20	0.16	0.24
Perceived remote teaching effectiveness^2^	0.44^*^	0.17	0.13	0.21
Education level handled^2^	0.32^*^	0.10	0.06	0.14
Activity evaluation^1^	0.30^*^	0.09	0.05	0.13
Initial confidence in ability to teach remotely^1^	0.29^*^	0.08	−0.01	0.07
Overly demanding expectations^1^	0.21^*^	0.05	0.01	0.09
Supportive teaching^1^	0.14^*^	0.02	−0.02	0.06
Reassuring attitude^1^	−0.12^*^	0.02	−0.02	0.06
Sense of competence^1^	0.10^*^	0.01	−0.03	0.05
Appraisal of relative situational impact	0.03	0.00	−0.04	0.04

* *Significant at p < 0.05*.

To reveal which of the indicators of teachers' professional adaptation to emergency remote instruction predict their perception of student coping with online classes (RQ4) and to find out the relative contribution of the respective predictors and the extent to which each of them determines teachers' perception of student coping (RQ5), we used ANCOVA to build a stepwise regression model with the forward selection procedure. It was preceded by a simple linear regression to additionally obtain a more general insight into how all the investigated indicators of teachers' professional and personal adaptation to ERT predict teachers' perception of student coping with online classes.

Table 4 illustrates the extent to which indicators of teachers' professional and personal adaptation to ERT predict their perception of student coping with online classes. The entire regression model is significant [*F*_(18, 1874)_ = 108.70, *p* < 0.001] and predicts ~51% of variance in teacher perception of student coping with remote learning.

**Table 4:** The regression results of the effects of indicators of teacher professional and personal adaptation to remote teaching on the perception of student coping with online learning.

**Dependent variable**	***R*^**2**^**	**Adj. *R*^**2**^**	***F***	**df1**	**df2**	**95%CI**	
Perceived student coping	0.51	0.51	108.70	18	1,874	0.48	0.53

To more deeply probe the relative role of the indicators, we built a multiple regression model based on forward selection using an ANCOVA design. The results are presented in Table 5. The teachers' perception of how students cope with remote learning depends most on teachers' evaluation uncertainty (β = −0.33, *t* = 17.19, *p* < 0.001) and instructional adjustment (β = −0.17, *t* = −8.96, *p* < 0.001). It is also predicted by the extent to which teachers feel able to evaluate students' activity (β = 0.14, *t* = 7.80, *p* < 0.001), followed by the mode of remote instruction (synchronous vs asynchronous; β = −0.10, *t* = −5.72, *p* < 0.001). The subsequent most consequential predictors turn out to be perception of the effectiveness of remote teaching (respectively less efficient than face-to-face instruction: β = −0.17, *t* = −9.45, *p* < 0.001 and equally efficient: β = 0.07, *t* = 3.90, *p* < 0.001), the education level handled (K-primary: β = −0.07, *t* = −4.23, *p* < 0.001 and tertiary: β = 0.10, *t* = 5.65, *p* < 0.001), immediately followed by teachers' appraisal of the relative impact of the pandemic situation on teachers and students (students affected more than teachers: β = −0.04, *t* = −2.38, *p* < 0.001 and both groups equally impacted by the pandemic context: β = 0.04, *t* = 2.46, *p* < 0.001). The subsequent predictors are supportive teaching (β = 0.11, *t* = 5.90, *p* < 0.001) and overly demanding expectations (β = 0.06, *t* = 3.57, *p* < 0.001) followed by sense of competence (β = −0.09, *t* = −4.36, *p* < 0.001) and perceived initial confidence in the ability to teach remotely (β = 0.09, *t* = 4.70, *p* < 0.001). The last two moderator variables are a reassuring attitude toward the students (β = −0.05, *t* = −2.60, *p* < 0.001) and prior experience with remote teaching (β = 0.04, *t* = 2.40, *p* < 0.001). Interestingly, teachers' gender did not contribute to the regression model.

**Table 5:** General linear model with ANCOVA (forward selection stepwise regression) for variables predicting teachers' perception of student coping with remote learning.

**Step**	**Independent variables**	***b***	**SE**	**β**	***t***	***R^**2**^***	**η_**p**_^2^**	**95%CI**		***F***
1	Evaluation uncertainty	−0.24	0.01	−0.33^*^	−17.19	0.30	0.14	0.11	0.16	295.61
2	Instructional adjustment	−0.15	0.02	−0.17^*^	−8.96	0.30	0.04	0.03	0.06	80.23
3	Activity evaluation	0.09	0.01	0.14^*^	7.80	0.21	0.03	0.02	0.05	60.88
4	Remote teaching mode (asynchronous)	−0.10	0.02	−0.10^*^	−5.72	0.14	0.02	0.01	0.03	32.73
5	Perceived remote teaching effectiveness Less efficient Equally efficient	−0.26 0.11	0.03 0.03	−0.17^*^ 0.07^*^	−9.45 3.90	0.23 0.06	0.05	0.03	0.07	48.19
6	Education level handled K-primary Tertiary	−0.15 0.18	0.03 0.03	−0.07^*^ 0.10 ^*^	−4.23 5.65	0.17 0.12	0.03	0.02	0.04	18.35
7	Appraisal of relative situational impact Students more affected than teachers Teachers and students affected equally	−0.08 0.07	0.03 0.03	−0.04^*^ 0.04^*^	−2.38 2.46	0.20 0.18	0.01	0.00	0.02	8.10
8	Supportive teaching	0.18	0.03	0.11^*^	5.90	0.31	0.02	0.01	0.03	34.76
9	Overly demanding expectations	0.04	0.01	0.06^*^	3.57	0.12	0.01	0.00	0.01	12.72
10	Sense of competence	−0.13	0.03	−0.09^*^	−4.36	0.36	0.01	0.00	0.0.02	19.01
11	Initial confidence in ability to teach remotely	0.06	0.01	0.09^*^	4.70	0.27	0.01	0.00	0.02	22.11
12	Reassuring attitude	−0.05	0.02	−0.05^*^	−2.60	0.20	0.00	0.00	0.01	6.74
13	Prior experience with distance teaching	0.05	0.02	0.04^*^	2.40	0.15	0.00	0.00	0.01	5.77
14	Gender	–	–	–	–		–			–

*b—unstandardized regression coefficient; SE—standard error; β—standardized regression coefficient*.

* *Significant at p < 0.001*.

## Discussion

The analyses revealed a number of factors that influenced teachers' perception of how their students were coping with emergency remote learning. These spanned both different aspects of professional adjustment and sociodemographic variables.

The inability to verify whether the students have been completing their online assignments and tests independently has been a recurrent concern, both in teachers' discussion forums and in the literature (e.g., Gonzales, 2020; Jargon, 2020). Given lack of an easy and foolproof way of ensuring student integrity outside of commercial automated proctoring software, many instructors have opted for open-book-style assessment that can both be individualized and at least does not penalize honesty.

The importance of being able to formatively evaluate students' activity is a related aspect. On the one hand, capacity to track students' progress facilitates summative evaluation at the end of the road. The divide created by the computer or smartphone screen impairs dialogue and the ability to provide tailored, ample feedback (Iwai, 2020) as well as to become aware of individual students' emotional and/or academic difficulties (Zaccoletti et al., 2020, p. 9). On the other hand, the outcome emphasizes the importance of teacher–student interaction. This connection is reinforced by the finding concerning the importance of synchronous classes, mirroring the results in Jelińska and Paradowski (2021). Limiting face-to-face interactions may adversely affect students' emotional development, emotional intelligence, and interpersonal and other soft skills (Hurst et al., 2013; Kaur and Bhatt, 2020, p. 45).

Teachers who felt their students were coping worse were at once more likely to have introduced major adjustments to their instruction. This is a logical connection, as such alterations and the reprioritization of curriculum goals (Reimers and Schleicher, 2020) are usually made in response to perceived problems.

Perceptions of student coping differed substantially depending on the education level handled. This finding is in line with observations by Hvas and Aller (2020) and Jelińska and Paradowski (2021). One possible explanation is that young learners are the most reliant on assistance, scaffolding, and support in their scholastic process (Paradowski, 2014, 2015), while parents and other guardians may not always be around during the pandemic—or be capable—to help out with technology, ensure that the children stay on task and submit their work in a timely manner, and help out in other ways necessary. Younger students are also more likely to be affected by contextual factors (Sameroff, 2010), are more vulnerable to the impact of traumatic experiences (Bartlett et al., 2020), and have been missing their other caregivers such as grandparents (Dalton et al., 2020), who play a major role in their lives (Salvador, 2008; Clemente-González, 2016). Older, less dependent students have better-developed self-regulation strategies and are therefore better able to adapt and take control over their learning (Herold, 2017; Zaccoletti et al., 2020, p. 9). Younger children are moreover less likely to have a computer/tablet/smartphone to access remote classes in real time; it is also much more difficult for them to have to spend long hours stationarily in front of the screen.

The findings also emphasized the importance of a supportive and reassuring attitude. Teacher support has been positively related to indicators of student behavioral engagement, including enhanced participation in school activities, heightened academic motivation, and depressed disruptive behaviors (Wang et al., 2013). Indeed, a humanistic approach and a pedagogy of compassion (Bozkurt and Sharma, 2020), while always crucial, gains particular importance in difficult times like these, when the psychological pressure of the lockdown, social distancing restrictions, and remote schooling pile on top of academic workload, homework (Commodari and La Rosa, 2020, p. 5), and online evaluations (Navarro-Mateu et al., 2020).

Finally, the results emphasize the significance of initial convictions. The importance of teachers' perception of the ease of use of technology in a high-school context had been underlined e.g. by Allen and Seaman (2013); in the context of the current pandemic, Lederman (2020) emphasized that a lot hangs on the initial implementation of ERT.

## Conclusions

The relationship between the student and the teacher has been claimed to be a major driver of the motivation to learn (Szabó, 2019, p. 19). Just as students' *self*-perceptions about own ability and competence are significantly associated with academic performance (Colom, 2012; De la Fuente et al., 2019; Ahmadi, 2020), *teachers'* perception of how their learners are coping have a strong automotive influence on their teaching effectiveness and may result in a self-fulfilling prophecy. Given that the post-pandemic world may see the trend of traditional classes becoming more blended (Kim, 2020) and increasingly integrating ICT (Gannon, 2019), there have been recommendations of teacher training to adjust to the new instructional format (Toquero, 2020). Awareness of the variables influencing teachers' perception of how the students are handling non-F2F instruction will be useful to both instructors themselves and program directors and may influence the content of helpful training and intervention programs. One such adjustment may be educators' heightened role as facilitators (remotely) guiding, monitoring, and motivating their students (Paradowski, 2015; Luthra and Mackenzie, 2020).

Given the short length of some of the scales, as well as the use of single-item indicators, further studies may be needed to verify the validity of the respective findings. Also, with the data analyzed in this contribution coming from a cross-sectional sample, one cannot confidently establish causal relationships. Complementary analyses from a longitudinal component of the survey will feature in future publications.

Another obvious limitation is the issue of respondent self-selection. Given that participation in this study was entirely voluntary and that not infrequently the questionnaire took upwards of 45 min to complete, the respondents were already motivated, could relate to the topic, and had the spare time and technology to comfortably fill it out. This means a limit on the representativeness and generalization potential of the data and resultant findings. Nonetheless, the robust effects of at least the most influential factors merit attention.

## Data Availability Statement

The raw data supporting the conclusions of this article will be made available by the authors, without undue reservation.

## Ethics Statement

The survey protocol had been approved by University of Warsaw's Human Research Ethics Committee. The respondents were provided with information about the survey and participated voluntarily.

## Author Contributions

MBP conceived the study and the questionnaire, piloted, and administered the data collection, performed the literature review, participated in the creation of the questionnaire and the writing of the manuscript. MJ carried out the analyses and participated in the creation of the questionnaire and the writing of the manuscript. All authors contributed to the article and approved the submitted version.

## Conflict of Interest

The authors declare that the research was conducted in the absence of any commercial or financial relationships that could be construed as a potential conflict of interest.

## Footnotes

^1^Jeliska, M., and Paradowski, M. B. (under review). The impact of demographics, life and work circumstances on college and university instructors' well-being during quaranteaching: findings from a global survey. *Front. Psychol*.

^2^The data presented in this paper come from a larger project https://schoolclosure.ils.uw.edu.pl/.

## Appendix

Jelińska, M. and Paradowski, M. B. (2021). Teachers' perception of student coping with emergency remote instruction during the COVID-19 pandemic: the relative impact of educator demographics and professional adaptation and adjustment. *Front. Psychol*. 12:648443. doi: 10.3389/fpsyg.2021.648443

**Table A1:** Instructional adjustment scale–means, standard deviations, and item-total score-corrected correlations of the items (*N* = 1, 944, α = .70, ω_h_ = 0.68, λ_6_ = 0.59, ρ = .98).

**Items**	**M**	**SD**	***r_***tt***_***
I have had to modify my lesson plans for remote teaching.	4.97	1.40	0.49
I have eased the grading scheme.	4.41	1.47	0.43
During this epidemic, I have felt that I have to alter not just the medium and method, but also the content of my classes.	4.29	1.60	0.54
**Scale**	13.66	3.55	0.41

**Table A2:** Evaluation uncertainty scale–means, standard deviations, and item-total score-corrected correlations of the items (*N* = 1,944, α = 0.73, ω_h_ = 0.75, λ_6_ = 0.68, ρ = 0.98).

**Items**	**M**	**SD**	***r_***tt***_***
I find it difficult to evaluate students' activity during online classes.	4.20	1.59	0.66
I am unable to verify whether the students are learning.	3.94	1.54	0.66
I am anxious that some students may be using the situation to cheat in their assignments/exams.	3.71	1.75	0.37
**Scale**	11.85	3.87	0.46

**Table A3:** Perceived student coping scale – means, standard deviations, and item-total score-corrected correlations of the items (*N* = 1,944, α = 0.72, ω_h_ = 0.74, λ_6_ = 0.69, ρ = 0.94).

**Items**	**M**	**SD**	***r_***tt***_***
I feel that some of my students have been left behind/fallen through the cracks in the shift to remote teaching.	2.48	1.51	0.53
My students have not coped well with remote learning.	3.42	1.39	0.67
My students have responded positively to my remote teaching.	4.23	1.18	0.37
The students have trouble with concentration and staying focused during the online classes.	2.83	1.45	0.48
**Scale**	12.97	4.10	0.40

**Table A4:** Supportive teaching scale–means, standard deviations, and item-total score-corrected correlations of the items (*N* = 1,944, α = 0.72, ω_*h*_ = 0.71, λ_6_ = 0.69, ρ = 0.73).

**Items**	**M**	**SD**	***r_***tt***_***
I can talk my students into learning.	4.69	1.01	0.38
I try to make sure everyone in a group feels included	5.07	0.86	0.53
I am good at helping people work well together.	4.84	0.92	0.53
I try to make my group members happy.	4.91	0.89	0.54
I trust my students.	4.67	0.95	0.39
**Scale**	24.18	3.16	0.34

**Table A5:** Sense of competence scale–means, standard deviations, and item-total score-corrected correlations of the items (*N* = 1,944, α = 0.76, ω_h_ = 0.76, λ_6_ = 0.74, ρ = 0.67).

**Items**	**M**	**SD**	***r_***tt***_***
I get things done quickly.	4.42	1.15	0.48
I know how to get things done.	4.88	0.92	0.55
Even with difficult tasks, I am always confident.	4.18	1.13	0.51
I like to work on tasks that require a great deal of skill.	4.64	0.99	0.45
I am a confident user of new technologies.	4.44	1.24	0.37
I continually train to keep my teaching skills and knowledge up-to-date.	4.56	1.18	0.39
I can manage many things at the same time.	4.87	1.07	0.52
I am always prepared.	4.65	1.10	0.39
**Scale**	36.61	5.25	0.29

**Table A6:** Results of exploratory factor analysis.

	**Factor 1**	**Factor 2**	**Factor 3**	**Factor 4**	**Factor 5**
My students have not coped well with remote learning.	0.26	−0.02	0.07	−0.31	0.79
I feel that some of my students have been left behind/fallen through the cracks in the shift to remote teaching. 0.23	0.01	0.03	−0.38	0.50
The students have trouble with concentration and staying focused during the online classes. 0.31	0.01	−0.01	−0.35	0.45
My students have responded positively to my remote teaching.	0.29	0.10	0.09	0.01	0.42
During this epidemic, I have felt that I have to alter not just the medium and method, but also the content of my classes. −0.02	0.03	0.08	0.69	−0.06
I have had to modify my lesson plans for remote teaching.	−0.16	0.02	0.03	0.68	−0.13
I have eased the grading scheme.	−0.22	0.01	−0.02	0.49	−0.10
I am unable to verify whether the students are learning.	−0.78	−0.11	−0.04	0.22	−0.21
I find it difficult to evaluate students' activity during online classes.	−0.71	−0.14	0.01	0.26	−0.15
I am anxious that some students may be using the situation to cheat in their assignments/exams. −0.45	−0.02	−0.10	0.02	−0.09
I try to make my group members happy.	0.02	0.15	0.72	0.00	0.00
I try to make sure everyone in a group feels included.	0.02	0.18	0.68	0.06	0.07
I am good at helping people work well together.	0.03	0.34	0.56	−0.02	0.03
I can talk my students into learning.	0.09	0.28	0.37	0.04	0.07
I trust my students.	0.16	0.24	0.35	0.01	0.10
I know how to get things done.	0.00	0.64	0.02	0.02	0.02
I get things done quickly.	0.02	0.60	0.15	0.10	0.00
I can manage many things at the same time.	−0.01	0.60	0.15	0.10	−0.01
Even with difficult tasks, I am always confident.	0.06	0.58	0.12	−0.07	0.01
I like to work on tasks that require a great deal of skill.	0.09	0.47	0.15	−0.05	0.04
I am always prepared.	−0.04	0.46	0.09	−0.04	0.01
I am a confident user of new technologies.	0.15	0.40	0.07	−0.05	0.03
I continually train to keep my teaching skills and knowledge up-to-date.	0.05	0.38	0.29	0.08	−0.03
